# The effect of adipose-derived stem cells (ADSC) treatment on kidney histopathological appearance on the Wistar rat models with grade five kidney trauma

**DOI:** 10.1515/iss-2023-0065

**Published:** 2024-07-24

**Authors:** Ahmad Agil, Tjahjodjati Romdam, Nur Atik, Dedi Rachmadi, Anglita Yantisetiasti, Ali E. Zumrutbas

**Affiliations:** Graduate School, Doctoral Program, Faculty of Medicine University of Padjadjaran, Bandung, Indonesia; Department of Urology, Faculty of Medicine University of Padjadjaran, Bandung, Indonesia; Department of Biomedical Sciences, Faculty of Medicine University of Padjadjaran, Bandung, Indonesia; Department of Pediatrics, Faculty of Medicine University of Padjadjaran, Bandung, Indonesia; Department of Anatomical Pathology, Faculty of Medicine University of Padjadjaran, Bandung, Indonesia; Department of Urology, Faculty of Medicine Pamukkale University, Denizli, Türkiye

**Keywords:** ADSC, fibrosis, kidney trauma, stem cells, histopathology, total renal score

## Abstract

**Objectives:**

Kidney trauma is the most common urological trauma. Technological advances have made conservative management possible for almost all kidney trauma. However, grade five kidney trauma needs to be carefully examined due to its various complications, especially late complications that often delayed in recognition thus forming irreversible morbidity, with the most common late complication is kidney damage due to ischemic and fibrotic process. This study aims to confirm the effect of Adipose-Derived Stem Cells (ADSC) on the prevention of fibrosis in grade five kidney trauma using Wistar rat models, where the fibrosis process will be measured with histopathological examination which had features of glomerular sclerosis, tubular atrophy, and interstitial fibrosis in kidney tissue, then followed by histopathological scoring and total renal score.

**Methods:**

A total of 22 adult rats were divided into five groups: one healthy control group, two trauma groups without ADSC, and two others trauma groups with ADSC. Two different treatment times were set: two weeks and four weeks after treatment. The data were tested for normality (Shapiro-Wilk test), while differences between groups were assessed using one-way ANOVA or Kruskal-Wallis test if the distribution was not normal.

**Results:**

For the result of total renal score, statistical analysis reveal a significant difference in the total renal score in the kidney trauma with ADSC group compared with kidney trauma without ADSC group in fourth week of observation (p=0.001).

**Conclusions:**

These findings highlighted ADSC capability to prevent fibrosis caused by grade five kidney trauma on the Wistar rat models, as proven by significantly reduced histopathological grading on fibrosis.

## Introduction

The incidence of urological trauma in abdominal trauma is about 10 % [1, 2]. Kidney trauma is the most common urological trauma, especially in adult men, with an incidence of around 1–5 % of all trauma cases and about 24 % of all solid organ trauma cases [1], [2], [3]. Based on its mechanism, kidney trauma is classified into blunt-force and sharp-force trauma.

About 80–85 % of kidney trauma is caused by blunt abdominal trauma, pelvic trauma, or back trauma [4]. Motor vehicle accidents, falls from heights, and beatings play a role in most blunt kidney trauma. High-speed deceleration can also be a cause of kidney trauma.

Over the past 20 years, advances in imaging and therapeutic approaches have increased the ability in kidney preservation and decreased the number of surgical interventions, thus preventing unnecessary nephrectomy. Therefore, most kidney trauma can now be managed conservatively [2, 5, 6].

Conservative management does have an advantage in terms of kidney preservation by preventing an unnecessary nephrectomy. However, we need to pay attention to grade five kidney trauma which managed conservatively, especially the one with the highest possibility of developing complications [2, 6]. In a grade five kidney trauma, which is the highest grade of kidney trauma, there are lacerations in several places that split the kidney (shattered kidney) [1, 2, 4]. Complications that occur can be early such as bleeding, infection, urine extravasation, urinoma, perirenal abscess, and sepsis. Whereas late complications can manifest as hydronephrosis, stone formation, chronic pyelonephritis, hypertension, Arteriovenous Fistula (AVF), pseudo-aneurysm, and kidney damage [1, 2]. In early complications, it is generally discovered immediately when the patient is still under hospital admission so that interventions can be performed as soon as possible. However, in late complications, due to its slow progressing process, it is often too late to be discovered, thus causing irreversible damage. The most common late complication is kidney damage [1, 2, 6].

This complication is closely related to the formation of fibrotic tissue in the kidney. In grade five kidney trauma, ischemia and infarction will occur in the affected part of the kidney, followed by apoptosis and fibrotic tissue formation. This formation process in the kidney is an irreversible pathological process that could resulte in kidney damage in the affected kidney. In a case report reported by Basil et al. in 2010, in patient with refractory hypertension due to late complication of kidney trauma, after nephrectomy and histopathological examination were performed, there was diffuse glomerulosclerosis with hyaline formation, thyroidization with hyalinization, and several blood vessel calcifications shown in the histopathological result [7].

In the process of biomolecular fibrosis formation, there is an overexpression of transforming growth factor-β1 (TGF-β1), an important mediator in fibrosis process and kidney damage, which also induces tissue inhibitors of matrix metalloproteinase-1 (TIMP-1) to inhibit matrix metalloproteinase (MMP), therefore blocking extracellular matrix (ECM) degradation. Furthermore, this process will stimulate fibroblasts to produce type I and type III collagen, thus resulting in excessive extracellular matrix deposit in the kidney known as the fibrosis process [8, 9].

From the conditions above, although conservative management in grade five kidney trauma has the advantage of renal preservation by preventing unnecessary nephrectomy, it turns out that this approach still has weaknesses with the possibility for complications, especially late complications, which is almost always too late to be found, and can result in irreversible morbidity. The problem, to date, is that there has been no proven therapy or research in preventing further complications of grade five kidney trauma managed conservatively.

With advances in regenerative medicine, stem cells therapy has been studied as a therapy for various medical conditions, but there have been no studies on the benefit of stem cells usage in kidney trauma. Stem cells originating from adipose tissue (adipose-derived stem cells/ADSC) are adult mesenchymal stem cells that are easily isolated. When influenced by the extracellular environment, it has an *in vitro* capacity to differentiate into other cell types, such as adipocytes, myocytes, osteoblasts, and neurons. These stem cells are known to produce diverse cytokines and growth factors, thus having cytoprotective effects that stimulate cell recovery. ADSC provides benefits for ease of harvesting, abundance source tissue, and inexpensive processing because no extensive culture is needed to get enough stem cells for treatment [10].

Based on previous literature studying ADSC effect on various tissue injuries, including kidney, this study aims to confirm the effect of ADSC on the prevention of ischemia and fibrosis process, also induce tissue repair and regeneration processes on grade five kidney trauma using the Wistar rat models, which can be indirectly proven by histopathological examination of the kidney which had features of glomerular sclerosis, tubular atrophy, and interstitial fibrosis, then followed by histopathological scoring and total renal score.

## Methods

### Animals

All animal procedures and tests were designed and carried out by considering their relevance to human or animal health, the advancement of knowledge, or the good of society, in accordance with those listed in the U.S. Government Principles for the Utilization and Care of Vertebrate Animals Used in Testing, Research, and Training [11]. This research was conducted after receiving approval and recommendations from Research Ethics Committee of Faculty of Medicine University of Padjadjaran (353/UN6.KEP/EC/2022).

The animal test subjects in this study were rats from the *Rattus norvegicus* species, Wistar strain, male sex, three months old, weighing 300–350 g and will be used as the subject of the grade five kidney trauma models.

The estimated sample size in this study uses Federer’s formula. Using this formula, a minimum sample size of five rats in each group was determined. The healthy control group consisted of two rats. The rationale for the smaller sample size in the healthy control group is that the usage of too many animals is against the principle of animal welfare.

### Study protocol

The rats were divided into five groups:

1. Group I (healthy control): Wistar rat that is not given any treatment.
2. Group II (trauma without therapy): Wistar rat modeled for grade five kidney trauma that is sacrificed at the second week.
3. Group III (trauma without therapy): Wistar rat modeled for grade five kidney trauma that is sacrificed at the fourth week.
4. Group IV (trauma with therapy): Wistar rat modeled for grade five kidney trauma that is given ADSC, and sacrificed at two the second week.
5. Group V (trauma with therapy): Wistar rat modeled for grade five kidney trauma that is given ADSC, and sacrificed at the fourth week.

In this study, two different treatment times were set. The first time point is two weeks after treatment, since the process of apoptosis in the kidney reaches the peak on the 15th day [12]. The second time point is 4 weeks after treatment because significant increase in collagen formation in the remodeling phase begin at this period [13].

The inclusion criteria in this study are male Wistar rats, weighing 300–350 g, are healthy and actively moving. Drop out criteria in this study were rats that die during the period of treatment, infection in the treatment area, and rats that have congenital abnormalities in the urinary tract that are found intraoperatively.

### ADSC cell production

#### Stem cells isolation from human adipose tissue (human ADSC)

Adipose tissue was obtained with a biopsy needle or liposuction aspiration, and then stored at room temperature. Adipose tissue was washed with phosphate-buffered saline (PBS) containing 5 % penicillin/streptomycin (P/S). During washing, the sample is stored in a sterile culture dish with type I collagenase 0.075 % prepared with a PBS containing penicillin/streptomycin 2 % for tissue processing [14].

Adipose tissue was thinly sliced using two scalpels, then it was put into a 25 or 50 mL pipette up and down several times to facilitate tissue processing. The sample is then incubated for 30 min at a temperature of 37°, 5 % CO_2_. Collagenase activity was neutralized with 5 mL of MEM which contains fetal bovine serum (FBS) 20 % to the tissue sample. The tissue was inserted into the pipette up and down several times to facilitate tissue disintegration [14].

The sample was centrifuged at a speed of 2,000 rpm for 5 min to get a stromal vascular fraction (SVF) containing ADSC. After centrifugation, it was shaken vigorously to combine pellets and cells. This step completes the separation of the stromal cells from the primary adipocytes. The centrifugation step was repeated. After centrifugation, solutions of collagenase on the top layer were aspirated without disturbing the cells. Suspension of pellets in a 1 mL lysis buffer were incubated for 10 min in ice and then washed with a 2 % PBS solution containing 2 % P/S. It was then centrifuged at a speed of 2000 rpm for 5 min. The supernatants were aspirated and resuspended in a maximum of 3 mL stromal medium added with 20 % FBS, 1 % l-glutamine, and 1 % P/S, and then the suspended cells were filtered through a 70 mm cell strainer. The cell-containing sample were placed in a culture dish coated in lysine and incubated at a temperature of 37° and 5 % CO_2_ [14].

#### Culture and expansion of ADSC

Seventy-two hours after being placed in the culture dish, all culture mediums were aspirated from the dish. The cells were washed with a warmed PBS and flushed by using a pipette over the cells several times to clean the cells entirely of tissue fragments and/or blood cells. Stromal medium was added as needed. Cells were maintained in a moist tissue culture incubator at 37° and 5 % CO_2_. The medium were changed every 2 days until the cell reaches a confluence rate of 80–90 % [14].

**Figure 1:**
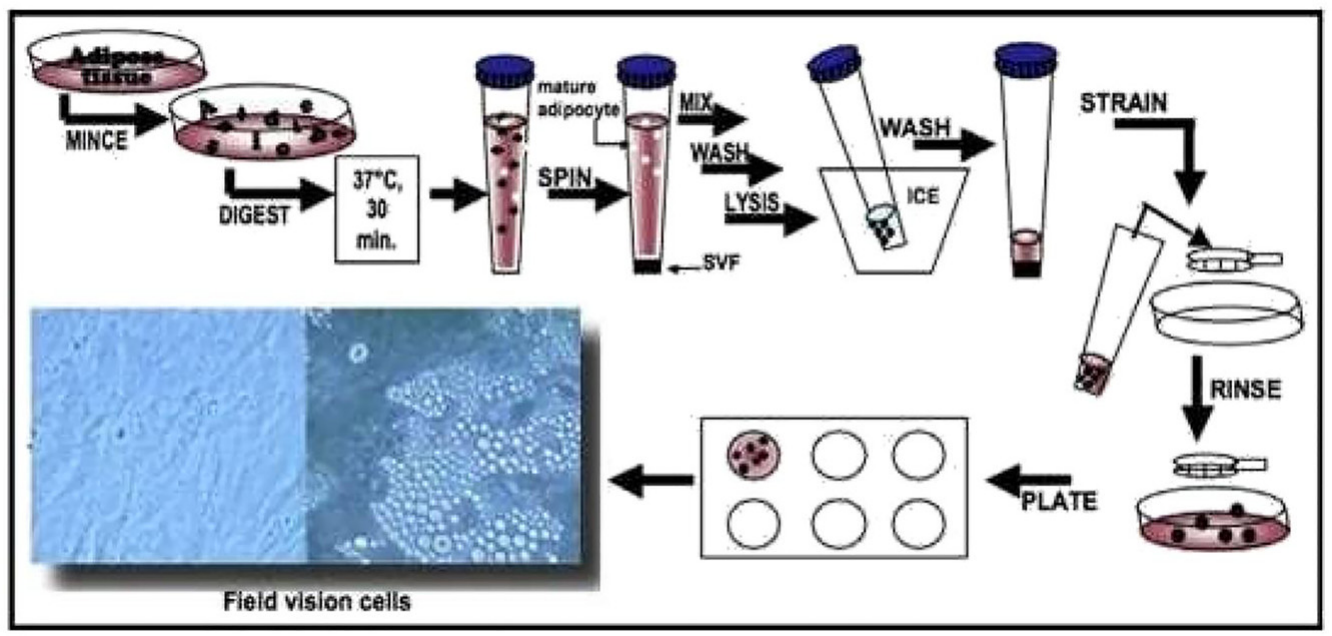
Processing fat cells into ADSC. Cited from: Bunnel BA et al. [14].

For vital ADSC retrieval, a small amount of sterile and warm PBS was added. PBS was replaced with a 0.5 % trypsin/EDTA solution. After 90 % of the cells have detached, 5 mL stromal medium were added to neutralize the trypsin reaction. The medium containing the suspended cells were moved from the culture dish into a 2 mL tube and centrifuged at 1,200 rpm for 5 min. Aspirate the supernatant and suspend the cells in small volumes of the medium stroma. Continue with the cell count by taking a small number of cells diluted in a trypan blue (for a solution of 1:2 add 12.5 ll suspended cells to 12.5 ll trypan blue). The cells were counted with a hemocytometer. The cell can then be placed back into the dish according to the capacity of the dish in the culture site. The medium is replaced 1 day after manufacture and then every 2 days (Figure 1) [14].

### Propagation of mesenchymal stem cells from results of fat tissue isolation

Mesenchymal stem cells that have successfully grown to form colonies can then be propagated until they reach the required dosage for clinical application purposes. The cells are then inserted into a sterile isotonic buffer with a ratio of 1 mL of fat tissue with 2 mL of collagenase for 2 h. Cells that have formed a monolayer coating with a confluence rate reaching 80 % need to be rejuvenated by performing passage. Passage was carried out by removing the medium from the petri dish and then rinsing the monolayer coating with a PBS solution. After that, the triple enzyme express was added and incubated for 5 min until the monolayer coating was detached from the Petri base. Passage was conducted six times, and then after the monolayer coating was detached, a medium stopper was added and resuspended until it turned into a single cell. The solution containing the single cell was poured into a conical tube and centrifuged until pellets were formed. The pellets were then delivered into MEM-ɑ and resuspended until it becomes a homogeneous solution and then planted in a new petri dish [15].

### Characterization of mesenchymal stem cells of fat tissue

**Figure 2:**
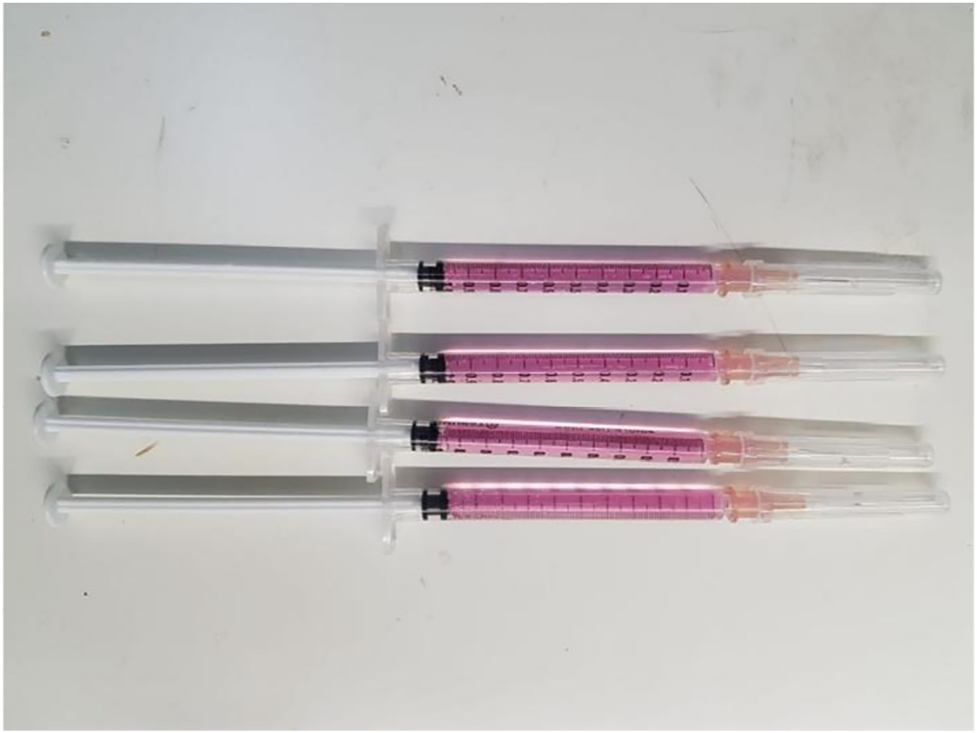
Ready to use human ADSC specimen.

Mesenchymal stem cells that have been successfully isolated from fat tissue were characterized using CD 105. CD 45, which is a specific marker of hematopoietic stem cells, were also used to ensure that the results of isolation from fat tissue are pure mesenchymal stem cells (Figure 2) [16]. In this study, we used human ADSC from ProStem PT. Prodia.

**Figure 3:**
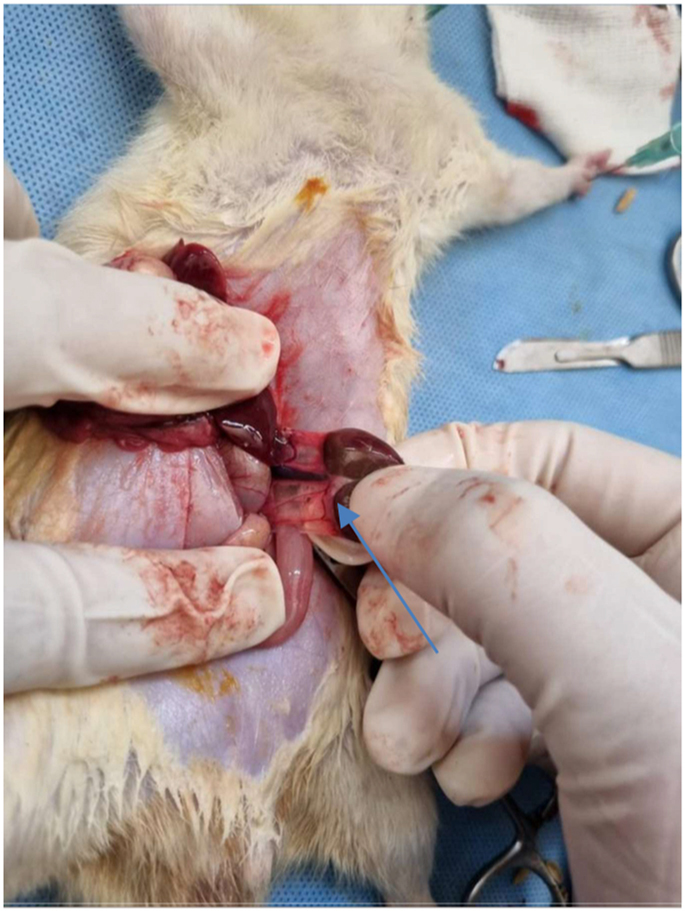
Midline incision and Wistar rat kidney (arrow).

### Methods of inducing kidney trauma

A total of 22 adult Wistar rats were included in the study. The cages were cleaned daily and the temperature was kept warm. After the same treatment for one-week, non-controlled samples (trauma group without therapy and trauma group with therapy) got several interventions to simulate grade five kidney trauma, with the steps described below:

1. Anesthesia was performed with intramuscular injection of ketamine HCl (25 mg/kg).
2. Rats were put in the supine position and sterilized with 10 % povidone-iodine; the operating field was covered with sterile doek.
3. Surgeons use sterile gloves and plastic covers, as well as face coverings (masks) from disposable paper.
4. Midline incisions were performed in the abdomen until the kidneys are exposed, then released.
5. A deep incision in the kidney was done to damage the pelviocalyceal system on the upper, middle, and lower pole, making sure the width of the incision reaches the lateral edge and medial edge. In the therapy group, ADSC injection was performed with a dose of 1 × 10^6^ cells through the peripheral vein access after the incision (Figure 3).
6. Surgical wound closure.
7. The skin was cleaned with 10 % povidone-iodine and the rats were placed in an enclosure with heating lamps.
8. All rats were given intramuscular ceftriaxone 30 mg/kg of body weight.
9. After a specified period (second week and fourth week), the rat from each group undergoes surgical removal of the kidney (nephrectomy).
10. After nephrectomy was complete, the rats were sacrificed.
11. Kidney tissue is examined for histopathological grading to assess the image of glomerular sclerosis, tubular atrophy and interstitial fibrosis using HE staining.

## Results

For histopathological examination, histopathological changes were measured in percentage of glomerular sclerosing appearance, percentage of atrophic tubules, and percentage of interstitial fibrosis per visual field. It was then scored based on the percentages as follows (Table 1): <10 % (0 point), 10–25 % (1 point), 26–50 % (2 point), and >50 % (3 point). The scores are then added to grade the overall severity of the chronic lesions (Table 2), into minimal (0–1 total score), mild (2–4 total score), moderate (5–7 total score), and severe (≥8 total score) [17].

**Table 1:** Scoring based on percentage per visual field appearance of glomerulosclerosis, tubular atrophy, and interstitial fibrosis.

Tissue appearance	Score			
	0	1	2	3
Glomerulosclerosis	< 10 %	10–25 %	26–50 %	>50 %
Tubular atrophy	< 10 %	10–25 %	26–50 %	>50 %
Interstitial fibrosis	< 10 %	10–25 %	26–50 %	>50 %

**Table 2:** Grading severity of lesion based on total renal score.

Grade	Total renal score
Minimal lesion	0–1
Mild lesion	2–4
Moderate lesion	5–7
Severe lesion	≥8

**Figure 4:**
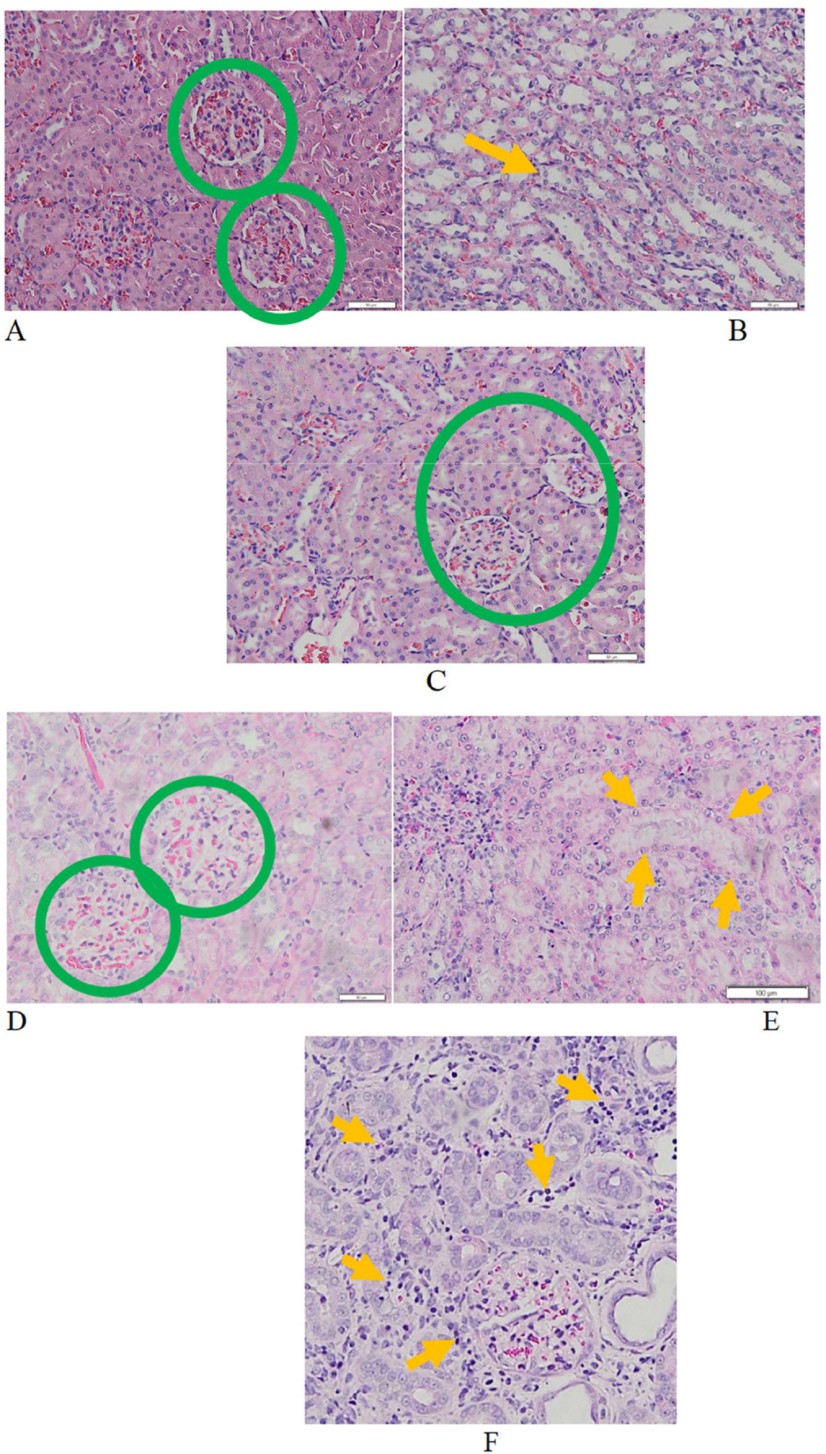

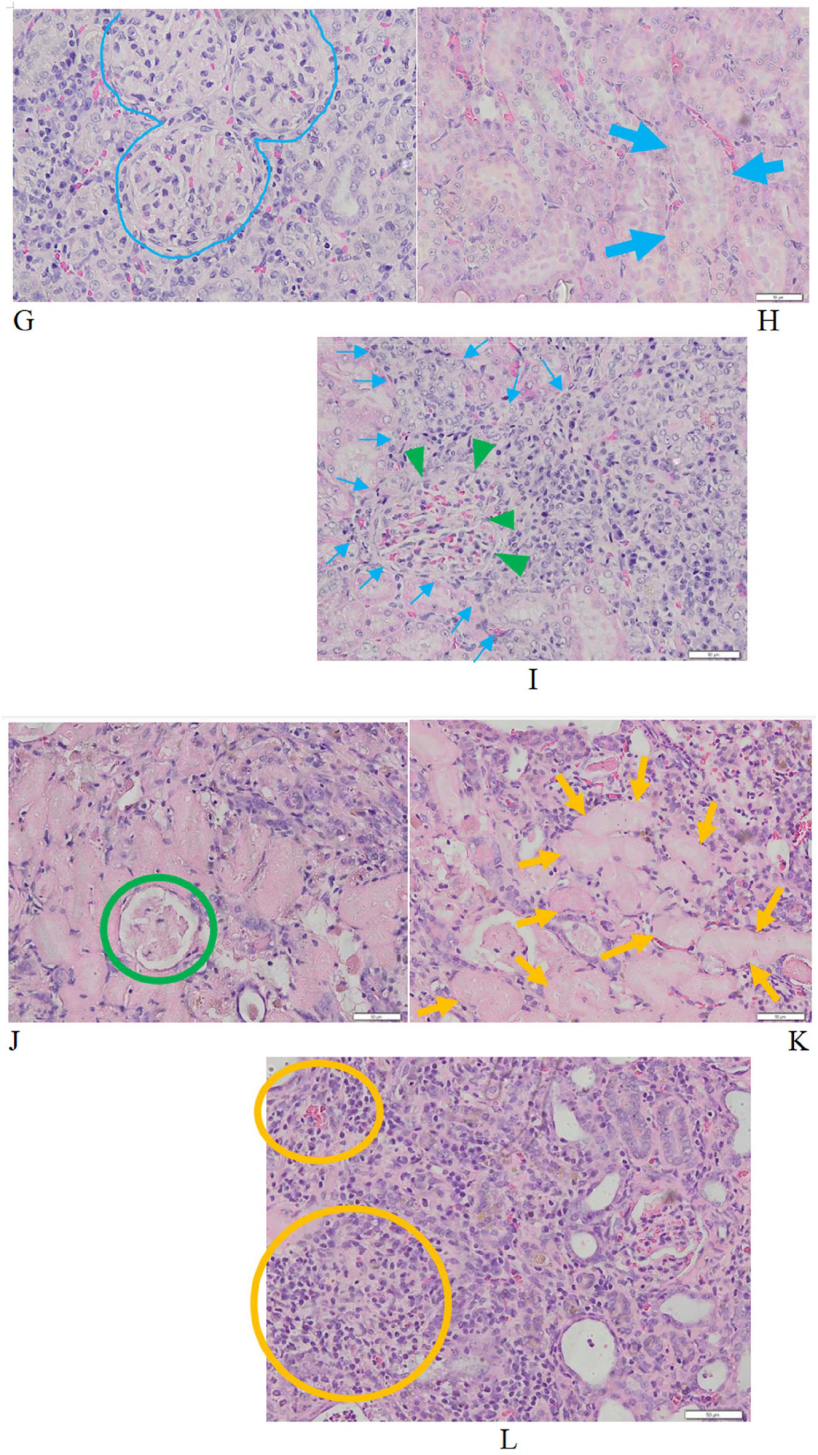
Kidney histhopathological features presenting fibrosis of glomerulus (glomerulosclerosis), tubulus (tubular atrophy), and interstitial tissue (interstitial fibrosis) (200x magnification). A. Normal glomerulus (score 0). Appearance of cappilaries and bowman space are normal (green circle). B. Normal tubules (score 0). The tubules are lined with cuboidal epithelium with good thickness and without any lumen dilatation (yellow arrow). C. Normal interstitial tissue (score 0). Interstitial tissue is the connective tissue between glomeruli and tubules, normally it is very few and thin. (Green circle). D. Glomerulosclerosis 10–25 % (score 1). There is extracellular matrix deposition in the glomerulus and bowman space narrowing with a total count of 10–25 % (green circle). E. Tubular atrophy 10–25 % (score 1). There are tubular cells flattening and lumen dilatation with a total count of 10–25 % (yellow arrow). F. Interstitial fibrosis 10–25 % (score 1). There are inflammatory cells infiltration in the interstitial connective tissue and fibrosis in the connective tissue between tubules with a count of 10–25 % (yellow arrow). G. Glomerulosclerosis 26–50 % (score 2). There is extracellular matrix deposition in the glomerulus and bowman space narrowing in three glomeruli with a total count of 26–50 % (blue circle). H. Tubular atrophy 26–50 % (score 2). There are flattening of the tubular cells and lumen diatation with a total count of 26–50 % (blue arrow). I. Interstitial fibrosis 26–50 % (score 2). It can be clearly seen there is infiltration of inflammatory cells in the interstitial connective tissue and fibrosis in the connective tissue between tubules and glomerulus (green arrow head) with a count of 26–50 % (area between blue arrows). J. Glomerulosclerosis >50 % (score 3, green circle). There is no longer appearance of normal glomerular structures on the specimen examined. K. Tubular atrophy >50 % (score 3). There are diffuse flattening of tubular cells and lumen dilatation in almost all tubules. (Yellow arrow). L. Interstitial fibrosis >50 % (score 3). It can be clearly seen there is massive infiltration of inlammatory cells scattered in the interstitial connective tissue and extensive fibrosis in the connective tissue between tubules and glomeruli with a count of >50 % (yellow arrow).

The results of histopathological examination in this study can be seen in Figure 4. In Figure 4 we can see kidney histhopathological features presenting fibrosis of glomerulus (glomerulosclerosis), tubulus (tubular atrophy), and interstitial tissue (interstitial fibrosis).

**Figure 5:**
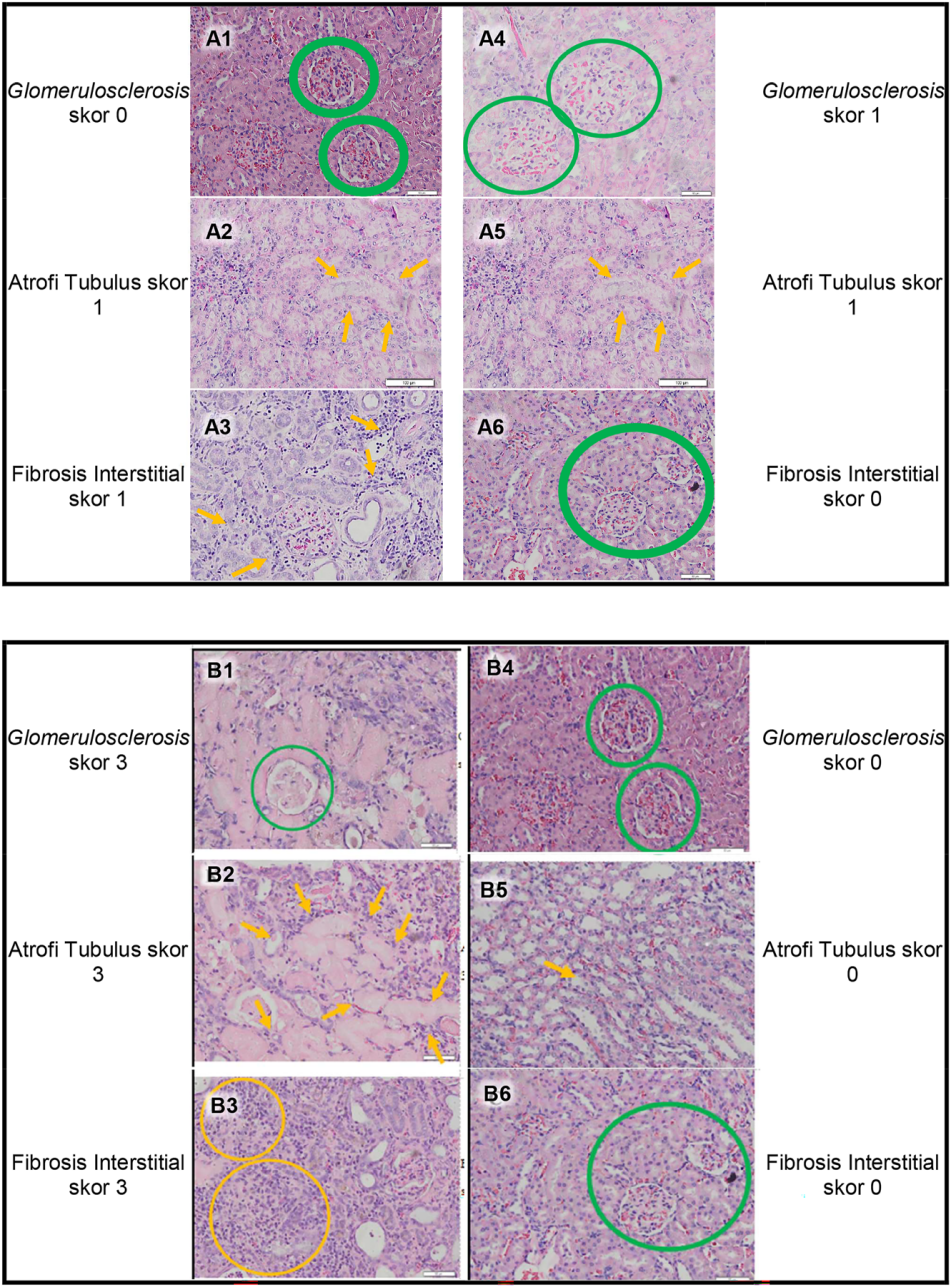
Representative feature of the total renal score which represents a feature of fibrosis in the kidney trauma group without ADSC therapy and the kidney trauma group with ADSC therapy in the second week and fourth week of observation. A1–3. Total renal score feature in the kidney trauma group without ADSC therapy in the second week of observation, with score 2 (mild lesion). A4–6. Total renal score feature in the kidney trauma group with ADSC therapy in the second week of observation, with score 2 (mild lesion). B1–3. Total renal score feature in the kidney trauma group without ADSC therapy in the fourth week of observation, with score 9 (severe lesion). B4–6. Total renal score feature in the kidney trauma group with ADSC therapy in the fourth week of observation, with score 0 (minimal lesion).

Furthermore, a representative feature of the total renal score is shown in Figure 5 which represents a feature of fibrosis in the kidney trauma group without ADSC therapy and the kidney trauma group with ADSC therapy in the second week of observation (Figure 5A) and the fourth week of observation (Figure 5B).

### Statistical analysis

The data was processed by SPSS version 20.0. The data was tested for normality (Shapiro-Wilk test). Differences between groups were assessed using one-way ANOVA (analysis of variance) statistical test, or Kruskal-Wallis tests if the distribution was not normal. The P-value which is considered significant is ≤0.05.

The results of statistical analysis on the research groups in histopathological examination seen on Table 3, obtained p values smaller than 0.05 (p value <0.05) in fourth week of observation (p=0.001), which means statistically significant. Thus it can be explained that there is a statistically significant difference in total renal score in fourth week of observation between kidney trauma with ADSC group and kidney trauma without ADSC group (Table 3).

**Table 3:** Comparative analysis of total renal score in second week and fourth week of observation.

Variable	Healthy control n=1	Kidney trauma without ADSC n=5	Kidney trauma with ADSC n=5	p-Value
**Total renal score second week** Mean±Std Median Range (min-max)	0.00 0.00 0.00	1.60 ± 1.517 2.00 0.00–3.00	2.80 ± 3.114 2.00 0.00–7.00	0.127
**Total renal score 4th week** Mean ± Std Median Range (min-max)	0.00 0.00 0.00	8.00 ± 1.732 9.00 5.00–9.00	0.00 0.00 0.00	0.001^a^

The sign^a^ indicates the value of p<0.05 means statistically significant.

## Discussion

In this study, grade five kidney trauma caused significant kidney injury and lead to severely fibrosis, as evidenced by grading severity of lesion based on total renal score in the kidney trauma group without ADSC. This study demonstrated that ADSC therapy markedly prevented renal fibrosis with a significant difference in total renal score in kidney trauma rat models with ADSC group compared with kidney trauma rat models without ADSC group in the fourth week of observation.

There was an article found that ADSC therapy could improve the histopathological appearance in the process of renal fibrosis due to unilateral partial obstruction of the ureteropelvic junction in the fourth week observation [18].

There is also another important article, in research with ischemic reperfusion injury in the kidney models, by administering ADSC in the early phase it was found that the process of renal fibrosis was minimal and the size of the kidneys remained normal compared to the group without therapy. In another article where a study was conducted in a group given ADSC in a late phase where it was estimated that significant fibrosis had occurred, it was found that there were also significant improvements compared to the group without therapy [19].

ADSC is known to have antifibrotic abilities by suppressing profibrotic mediator TGF-β1 as well as producing various secretomes such as vascular endothelial growth factor (VEGF), hepatocyte growth factor (HGF), granulocyte-macrophage stimulating factor (GM-CSF), basic fibroblast growth factor (bFGF), brain derived neurotrophic factor (BDNF), insulin growth factor-1 (IGF-1) and interleukins such as IL-6, IL-8, IL-10 which contribute in restoring the microenvironment in the tissue occupied by ADSC, thereby inhibiting the formation of fibrotic tissue [20].

In accordance with this study, recent studies have also found that ADSC treatment significantly reduces renal fibrosis and reduces renal levels of mRNA COL1A1, TGFB1, CTGF, and ACTA2. Some studies have used ADSC as a therapeutic strategy in the kidney injury due to ischemia, folic acid nephrotoxicity, or nephrectomy to inhibit damage [21], [22], [23]. The beneficial effects described include reduced renal fibrosis and chronic inflammation; indicated by reduced deposition of interstitial collagen, tissue chemokines, and cytokine expression. In the study of Furuichi et al. repeated administration of ADSC reduced acute tubular necrosis and infiltration of interstitial macrophages in injured kidneys and reduced the expression of cytokines and chemokines [23]. Other reported effects included a reduction in plasma creatinine levels and improved kidney function [24].

Research has also shown that systemic application of ADSC results in improvements in renal fibrosis, reduces the amount of fibrotic tissue, improves kidney function, and reduces gene expression of profibrogenic molecules [24]. In addition, the antifibrogenic effects of mesenchymal stem cells have been reported in various organs such as the lungs, liver, and kidneys [25–28].

Another study conducted by Chen et al. demonstrated that ADSC therapy minimized kidney injury following IRI by suppressing oxidative stress and the inflammatory response [28]. Furuichi et al. also showed that ADSC were able to ameliorate AKI induced secondary to IRI in a mouse model via the suppression of cytokines (IL-1β and TNF-α) and chemokines (macrophage inflammatory protein-1α), which led to an anti-inflammatory activity and alleviation of tubular necrosis [29].

ADSC also have late regenerative stimulant activity. Sheashaa et al. showed a markedly significant improvement in the creatinine clearance on day-14 after ADSC treatment without a proportional reduction of the tissue malondialdehyde level, which served as an indicator of oxidative stress [30].

In conclusion, the results of this study demonstrate the ability of ADSC to prevent fibrosis caused by grade five kidney trauma on the Wistar rat models, as proven by significantly reduced histopathological grading on fibrosis. The mechanism hypothesized is due to early protective activity and late regenerative stimulant activity of ADSC in preventing fibrosis process.
